# Host-directed therapy in foals can enhance functional innate immunity and reduce severity of *Rhodococcus equi* pneumonia

**DOI:** 10.1038/s41598-021-82049-y

**Published:** 2021-01-28

**Authors:** Angela I. Bordin, Noah D. Cohen, Steve Giguère, Jocelyne M. Bray, Londa J. Berghaus, Brenton Scott, Rena Johnson, Magnus Hook

**Affiliations:** 1 Equine Infectious Disease Laboratory, Department of Large Animal Clinical Sciences, College of Veterinary Medicine & Biomedical Sciences, Texas A&M University, College Station, TX USA; 2 Department of Large Animal Medicine, College of Veterinary Medicine, University of Georgia, Athens, GA USA; 3 Pulmotect, Inc., Houston, TX USA; 4 Institute of Biosciences and Technology, Center for Infectious and Inflammatory Diseases, Texas A&M University, Houston, TX USA

**Keywords:** Immunology, Cytokines, Infection, Infectious diseases, Innate immune cells, Innate immunity, Translational immunology

## Abstract

Pneumonia caused by the intracellular bacterium *Rhodococcus equi* is an important cause of disease and death in immunocompromised hosts, especially foals. Antibiotics are the standard of care for treating *R. equi* pneumonia in foals, and adjunctive therapies are needed. We tested whether nebulization with TLR agonists (PUL-042) in foals would improve innate immunity and reduce the severity and duration of pneumonia following *R. equi* infection. Neonatal foals (n = 48) were nebulized with either PUL-042 or vehicle, and their lung cells infected ex vivo. PUL-042 increased inflammatory cytokines in BAL fluid and alveolar macrophages after ex vivo infection with *R. equi*. Then, the in vivo effects of PUL-042 on clinical signs of pneumonia were examined in 22 additional foals after intrabronchial challenge with *R. equi*. Foals infected and nebulized with PUL-042 or vehicle alone had a shorter duration of clinical signs of pneumonia and smaller pulmonary lesions when compared to non-nebulized foals. Our results demonstrate that host-directed therapy can enhance neonatal immune responses against respiratory pathogens and reduce the duration and severity of *R. equi* pneumonia.

## Introduction

*Rhodococcus equi* is a facultative, intracellular bacterium that preferentially replicates within macrophages^1^ and causes a pyogranulomatous pneumonia in foals and immunocompromised people that resembles tuberculosis. Infection of foals with *R. equi* occurs during the first weeks after birth^2^, when foals are more susceptible to infection with this pathogen^3^, probably because of the naivety/immaturity of both innate and adaptive immune responses^4–9^. Adaptive immune responses require more time to develop such that neonates of all species have a window during which they are not able to be protected by vaccines and consequently depend heavily on innate immune responses^10^. Even if foals could generate an effective adaptive immune response to vaccination, no vaccine against *R. equi* pneumonia is commercially available despite decades of research. There is evidence that maternal vaccination against *R. equi* protects foals against clinical pneumonia^11^; that approach, however, has some limitations such as it is dependent on the dam’s immune status and adequate immune response to vaccination, ingestion of sufficient colostrum in the first 24–36 h after birth (there is no transfer of immunoglobulins through the placenta in the equine species), etc. Other preventive methods such as transfusion of *R. equi*-specific hyperimmune plasma are not completely effective and carry some risks for foals^12–15^. Chemoprophylaxis with macrolides or other classes of antimicrobials is not recommended because of emerging resistance of *R. equi* (and other bacteria) to these drugs^16^. Thus, control of *R. equi* pneumonia in foals relies on antimicrobial treatment of foals affected with either clinical or subclinical pneumonia^17^.

Macrolides have been the standard antibiotic used for decades to treat *R. equi*^16^. Resistance to macrolides in *R. equi* is an emerging problem in equine medicine that has been linked to the practice of widespread antimicrobial treatment of foals with subclinical pneumonia at farms with endemic *R. equi*^17–22^. Because effective alternative antimicrobials for treating foals with *R. equi* infections are limited^18,23–26^, there is an urgent need for non-antibiotic approaches such as host-directed therapy (HDT) to fight these pathogens. Immunotherapy targeting innate immune responses in neonates is an important strategy for HDT because the immune system of foals is immature^4–9^ and infection with *R. equi* appears to occur early in life^2^ when foals are known to be more susceptible to this bacterium^3^.

Toll-like receptors (TLRs) are among the principal signaling receptors for initiating innate immunity, resulting in various effects including cytokine production^27^. The intracellular TLR9 recognizes bacterial unmethylated cytosine-phosphate-guanine oligodeoxynucleotides (CpG-ODNs)^28^. We have previously demonstrated that a B class CpG-ODN induced expression of pro-inflammatory cytokines by immune cells of foals both ex vivo and in vivo^4,29–31^, including interferon-gamma (IFN-γ). Evidence exists that younger foals have lower levels of IFN-γ at birth than older foals^7,8^, and that stimulation of phagocytes with IFN-γ promotes killing of intracellular bacteria such as *R. equi*^32,33^ and *Mycobacterium tuberculosis* (Mtb)^34^. The TLR2 recognizes lipoteichoic acid and peptidoglycan of Gram + bacteria. TLR2^−/−^ mice are more susceptible to both *R. equi* infection^35^ and Mtb^36^ indicating that activation of the TLR2 signaling pathway can be important in defense against intracellular bacteria. PUL-042 (Pulmotect, Inc., Houston, TX) is an inhaled product that combines two synthetic TLR agonists, Pam2CSK4 (ligand of TLR2/6) and a class C CpG-ODN M362 (ligand of TLR9). This compound drug has been tested in many species against different viral, bacterial, and fungal pathogens and has shown promising results of increasing host survival and reducing pathogen burden^37–42^.

The objective of our research was to test whether nebulization with PUL-042 in foals would improve innate immune responses and reduce the severity and duration of pneumonia due to intrabronchial challenge with *R. equi* of foals at 28 days of age. We demonstrate that: (1) a combination of TLR2/6 and TLR9 agonists (PUL-042) administered via nebulization at various doses and on multiple occasions is safe for neonatal foals; (2) aerosol treatment of neonatal foals with PUL-042 increased interleukin (IL)-6 and IFN-γ concentration in the supernatant of alveolar macrophages (AMs) upon ex vivo infection; (3) nebulization of PUL-042 in foals challenged intrabronchially with live virulent *R. equi* reduced the duration of fever, clinical pneumonia, and cough, as well as reduced the size of pulmonary lesions compared to foals that were not nebulized. Our results indicate that nebulization with PUL-042 enhanced functional responses by AMs and reduced the severity of clinical pneumonia compared to non-nebulized foals.

## Results

### Nebulization with PUL-042 is safe for newborn foals

**Figure 1:**
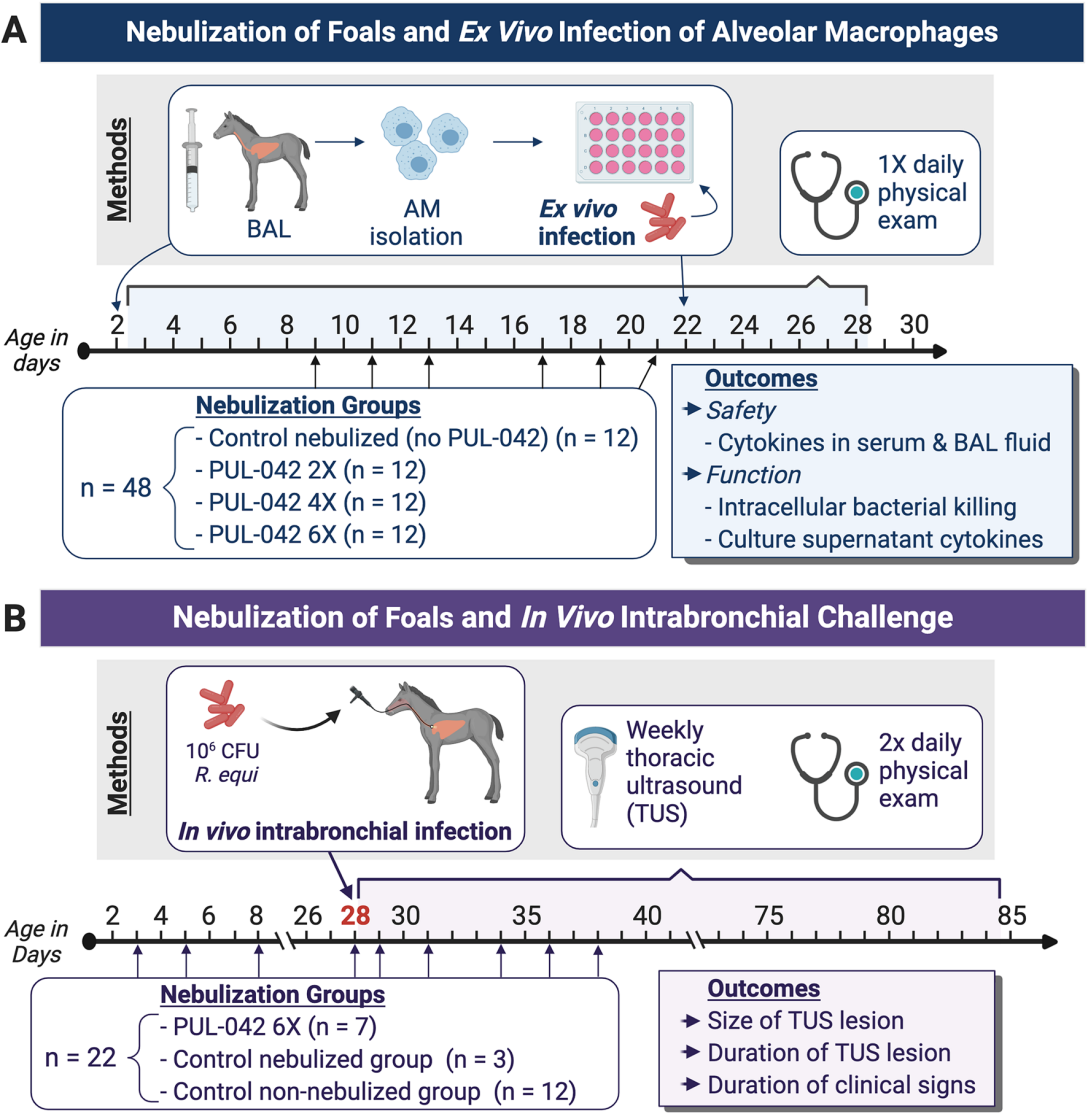
Study design. (**A**) Ex vivo study; All foals in this study were nebulized with either PUL-042 or placebo, and *Rhodococcus equi* infections occurred ex vivo in alveolar macrophages (AMs) obtained through broncho-alveolar lavage (BAL). Control group (n = 12) received 2.8% glycerol, the vehicle diluent for PUL-042; PUL-042 2× (n = 12) received 46 µg Pam2 and 68 µg ODN; PUL-042 4× (n = 12) 92.8 µg Pam2 and 136 µg ODN; PUL-042 6× (n = 12) 139.8 µg Pam2 and 204 µg ODN. BAL procedures for collection of fluid and AM were performed on ages 2 (before nebulization) and 22 days (24 h following the last nebulization). Nebulizations started 1 week after the first BAL (approximately day 9 of age), and each foal received 6 nebulizations in a 2-week interval. Infections were performed ex vivo in AMs. (**B**) In vivo study; PUL-042 6× (n = 7) received 139.8 µg Pam2 and 204 µg ODN; control-nebulized group received 2.8% glycerol (n = 3); and control non-nebulized group (n = 12) did not receive nebulization. Infections were performed in vivo on day 28 of age, and foals were in the study until approximately 12 weeks of age. Nebulized foals received 9 nebulizations: 4 before and 5 after challenge at 28 days of age. Following challenge, twice daily physical examination, and weekly thoracic ultrasounds (TUS) were performed in all foals. At 12 weeks of age or whenever the foal recovers from clinical pneumonia if after 12 weeks of age, a tracheal wash was performed to confirm complete recovery of pneumonia by demonstrating absence of cultured *R. equi* and evidence of normal cytology on tracheal-wash fluid. Created with BioRender.com.

**Figure 2:**
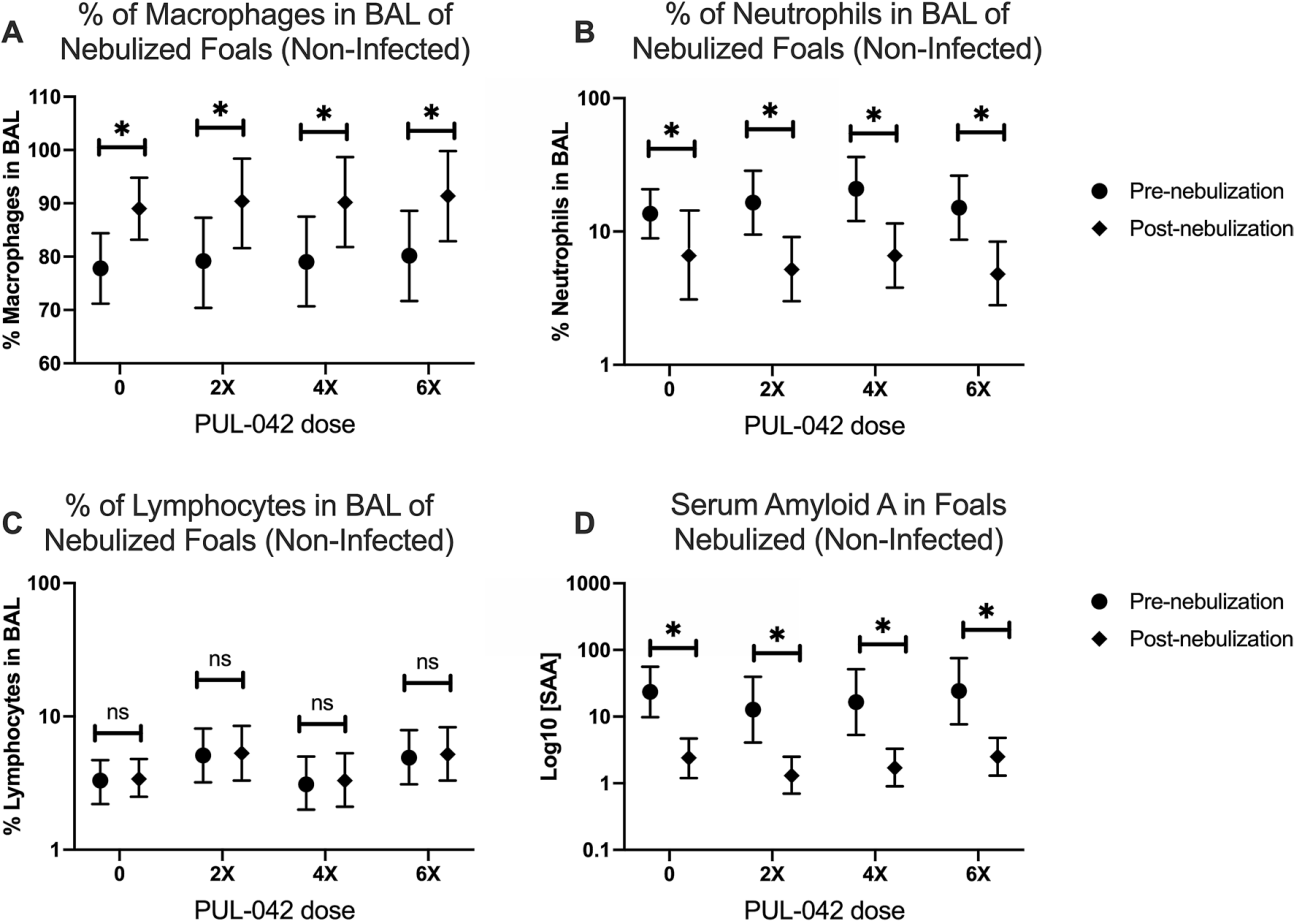
Broncho-alveolar lavage (BAL) fluid differential cytology (**A**–**C**) and serum amyloid A (SAA; (**D**)) of foals either before (Pre-nebulization) or after (Post-nebulization) serial nebulization with PUL-042 at various concentrations: 0 (n = 12), 2× (n = 12), 4× (n = 12), or 6× (n = 12). All foals were nebulized and no foals were infected in vivo with *Rhodococcus equi*. BALs and blood collection were performed pre- (day 2 after birth) and post-nebulization (day 22, 1 day after the last nebulization). Mean is represented by the symbol (circle for pre-nebulization; diamond for post-nebulization) and 95% confidence interval (whiskers). Percentage of macrophages (**A**) in BAL increased significantly (P < 0.0001) while percentage of neutrophils (**B**) decreased (P < 0.0001) after treatment (post) in all groups. The percentage of lymphocytes (**C**) did not vary significantly. Serum amyloid A (**D**) significantly decreased post-nebulization in all groups irrespective of treatment (P < 0.0001).

**Figure 3:**
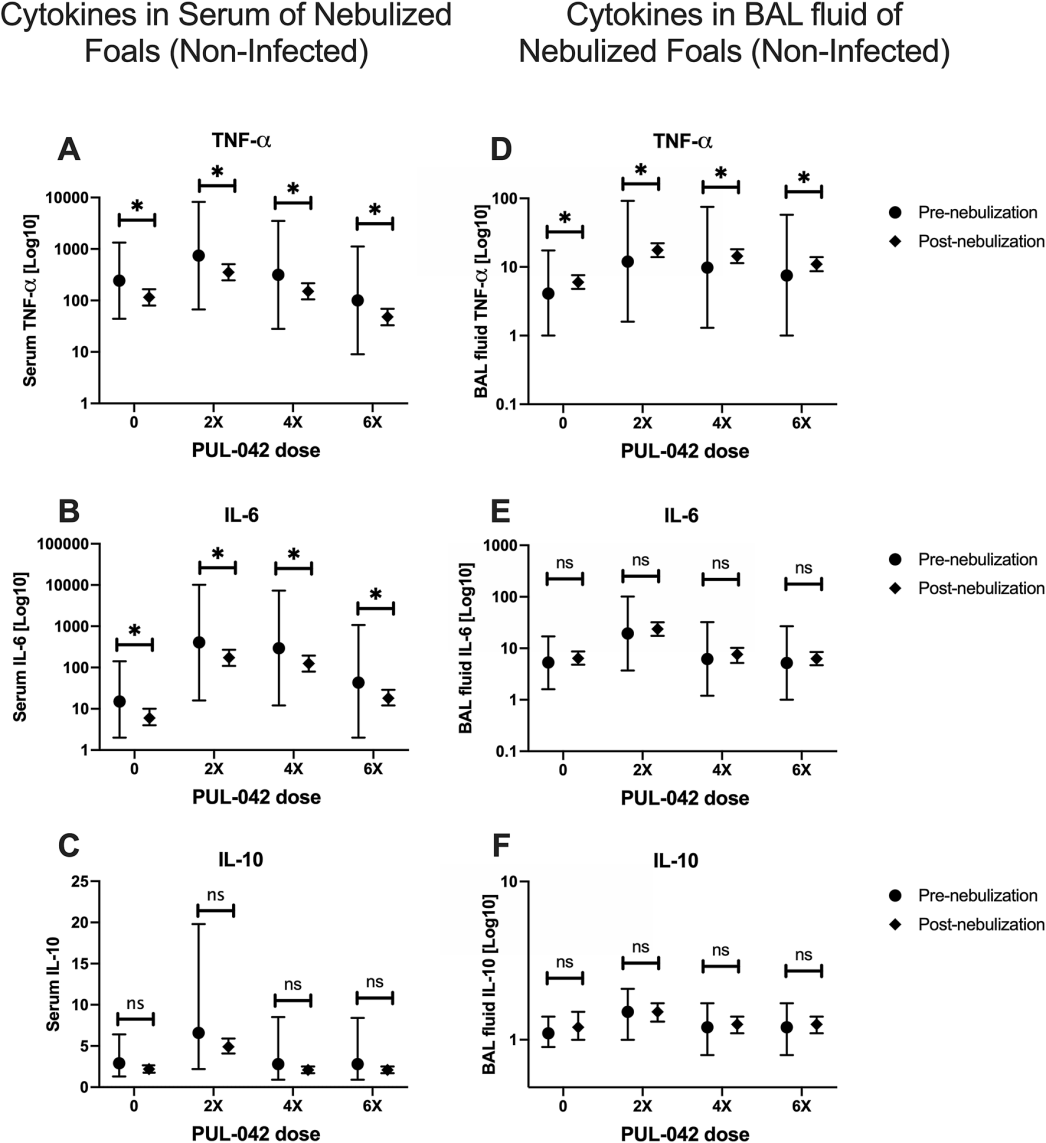
Concentration of pro- and anti-inflammatory cytokines in serum (left panel) or broncho-alveolar lavage (BAL; right panel) fluid of foals either before (Pre-nebulization) or after (Post-nebulization) serial nebulization with PUL-042 at various concentrations: 0 (n = 12), 2× (n = 12), 4× (n = 12), or 6× (n = 12). Cytokines were measured per ml of either serum or BAL fluid (TNF-α = pg/ml; IL-6 = pg/ml; IL-10 = ng/ml). All foals were nebulized and no foals were infected in vivo with *Rhodococcus equi*. BALs were performed pre (day 2 after birth) and post (day 22, 1 day after the last nebulization). In serum, TNF-⍺ (**A**) and IL-6 (**B**) significantly decreased with age, but only IL-6 had 1 group (PUL-042 2X) that was significantly different than PUL-042 0. Serum concentration of IL-10 (**C**) did not significantly differ among treatment groups or age. Tumor necrosis factor-alpha significantly increased in BAL fluid (**D**) for all groups, but effects were higher in BAL fluid of PUL-042 treated animals. The concentration of IL-6 (**E**) and IL-10 (**F**) in BAL fluid also did not significantly change with either age or treatment. Mean is represented by the symbol (circle for pre-nebulization; diamond for post-nebulization) and 95% confidence interval (whiskers).

Forty-three foals (36 foals from ex vivo study, seven from in vivo challenge study; Fig. 1A,B) were nebulized multiple times with PUL-042 in this study, and none had any detectable adverse reaction to the nebulization such as increase in respiratory effort and rate, lacrimation, cough, nasal discharge, etc. The percentage of macrophages significantly increased (Fig. 2A) and the percentage of neutrophils decreased (Fig. 2B) with age in BAL cells in all foals, irrespective of treatment. The percentage of lymphocytes did not change with age or treatment group (Fig. 2C). There was no indication of systemic inflammation attributable to nebulization with PUL-042. Serum amyloid A (Fig. 2D), IL-6 (Fig. 3A) and TNF-α (Fig. 3B) decreased with age for all groups independent of their aerosolization treatment. Interleukin-10 concentrations in serum were not altered by nebulization (Fig. 3C). Plasma fibrinogen concentration did not change significantly following treatment (data not shown). These results indicated that nebulization with PUL-042 was safe for newborn foals and did not induce either systemic or local inflammation.

### Nebulization with PUL-042 influences cytokine production in BAL fluid and AMs infected ex vivo with *R. equi*

We performed BALs in 48 foals before and after six nebulizations in the second and third week after birth with either 2.8% glycerol or one of three different doses of PUL-042, and collected both BAL cells and fluid (Fig. 1A). Foals nebulized with PUL-042 doses 2× and 4× had increased TNF-α in BAL fluid compared with foals receiving glycerol (Fig. 3D), but no other cytokine (IL-6, IL-10; Fig. 3E,F) concentrations in BAL fluid were altered by treatment. Interleukin-1α and IL-1β were not detected in BAL fluid from foals at either time-point and were not included in the analysis.

**Figure 4:**
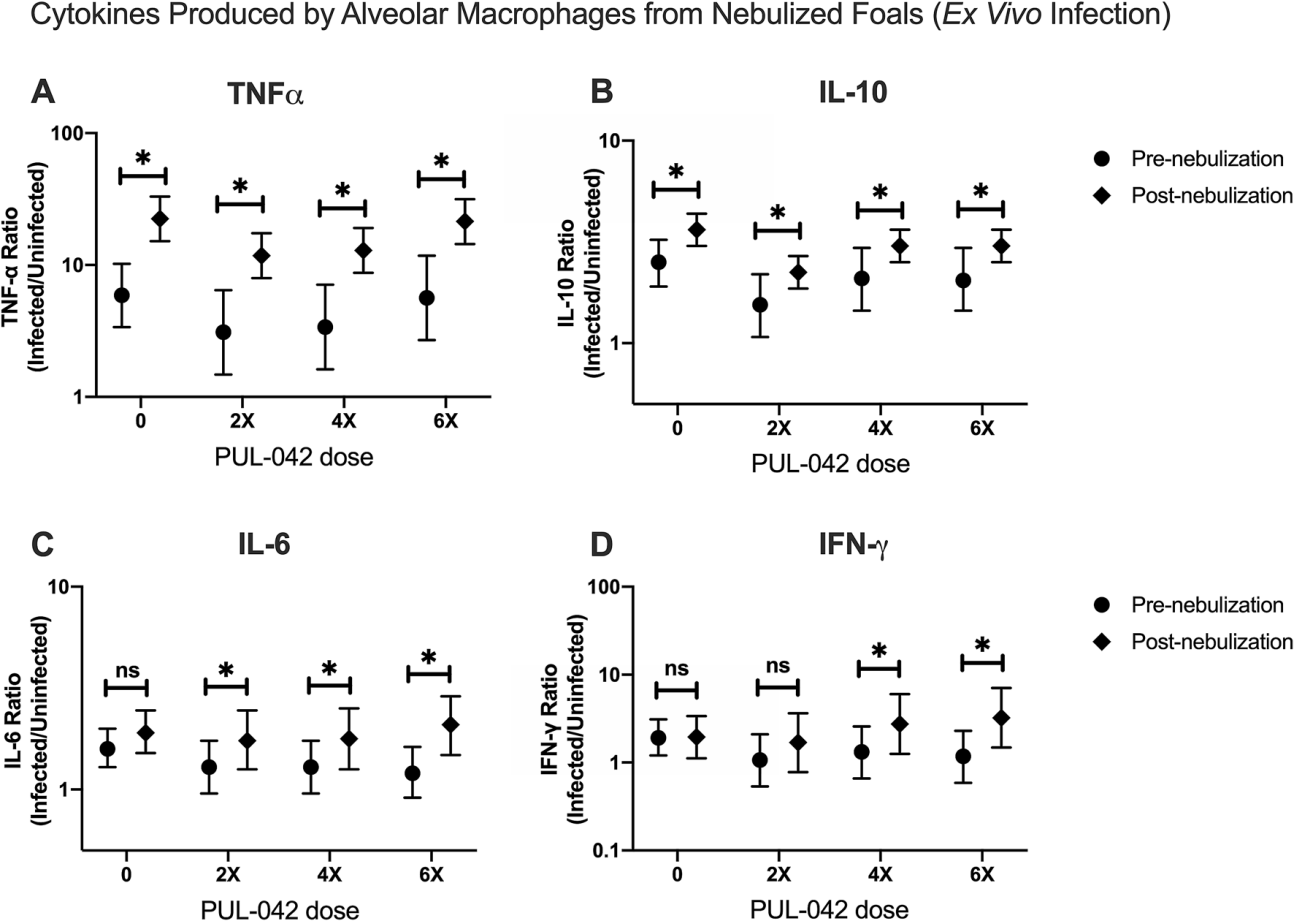
Concentration of pro- and anti-inflammatory cytokines in supernatants obtained from cultured alveolar macrophages (AMs) from foals before (age 2 days; Pre-nebulization) and after (age 28 days; Post-nebulization) nebulization with glycerol 2.8% (PUL-042 0; n = 12), and varying doses of PUL-042 (2X, n = 12; 4×, n = 12; 6×, n = 12). All foals were nebulized and no foals were infected in vivo with *Rhodococcus equi*. Broncho-alveolar lavage was performed pre- (day 2 after birth) and post-nebulization (day 22, 1 day after the last nebulization): AMs were infected with virulent *R. equi* using a multiplicity of infection of 10 bacteria per macrophage (infected) or kept in media only (uninfected). Supernatant was collected 48 h post-infection for determination of cytokine concentration by ELISA. Data represented as estimates of mean (95% CI); values were analyzed as ratios of *R. equi*-infected/uninfected cells to account for cytokine production at baseline (control uninfected). Upon ex vivo infection with *R. equi*, both cytokines TNF-⍺ (**A**) and IL-10 (**B**) ratios significantly increased for all groups post-nebulization, while IL-6 ratio significantly increased only after treatment with PUL-042 (**C**). For INF-γ, this significant increase was only observed for the 2 higher doses of PUL-042, 4× and 6× (**D**).

Alveolar macrophages from nebulized foals were collected and either kept in media (uninfected, baseline) or infected ex vivo with *R. equi* (Fig. 1A). The ratio of the concentration of infected/uninfected wells were used because of the high inter-individual variation in baseline production of cytokines. Interleukin-10 and TNF-α in infected cells increased relative to uninfected AMs in all treatment groups including the control foals (Fig. 4A,B), suggesting an age-related increase in response to infection. The AMs from foals nebulized with the two highest doses of PUL-042 that were infected ex vivo with *R. equi* produced greater IL-6 (Fig. 4C) and IFN-γ compared to cells from foals nebulized with 2.8% glycerol alone (Fig. 4D), indicating PUL-042 resulted in an increased immune response to infection but did not alter responses in uninfected cells. Interleukin-1α and IL-17 were not detected in the supernatant of cultured AMs at either time-point and were not included in the analysis.

### Nebulization with PUL-042 does not improve ex vivo killing of *R. equi* by AMs

**Figure 5:**
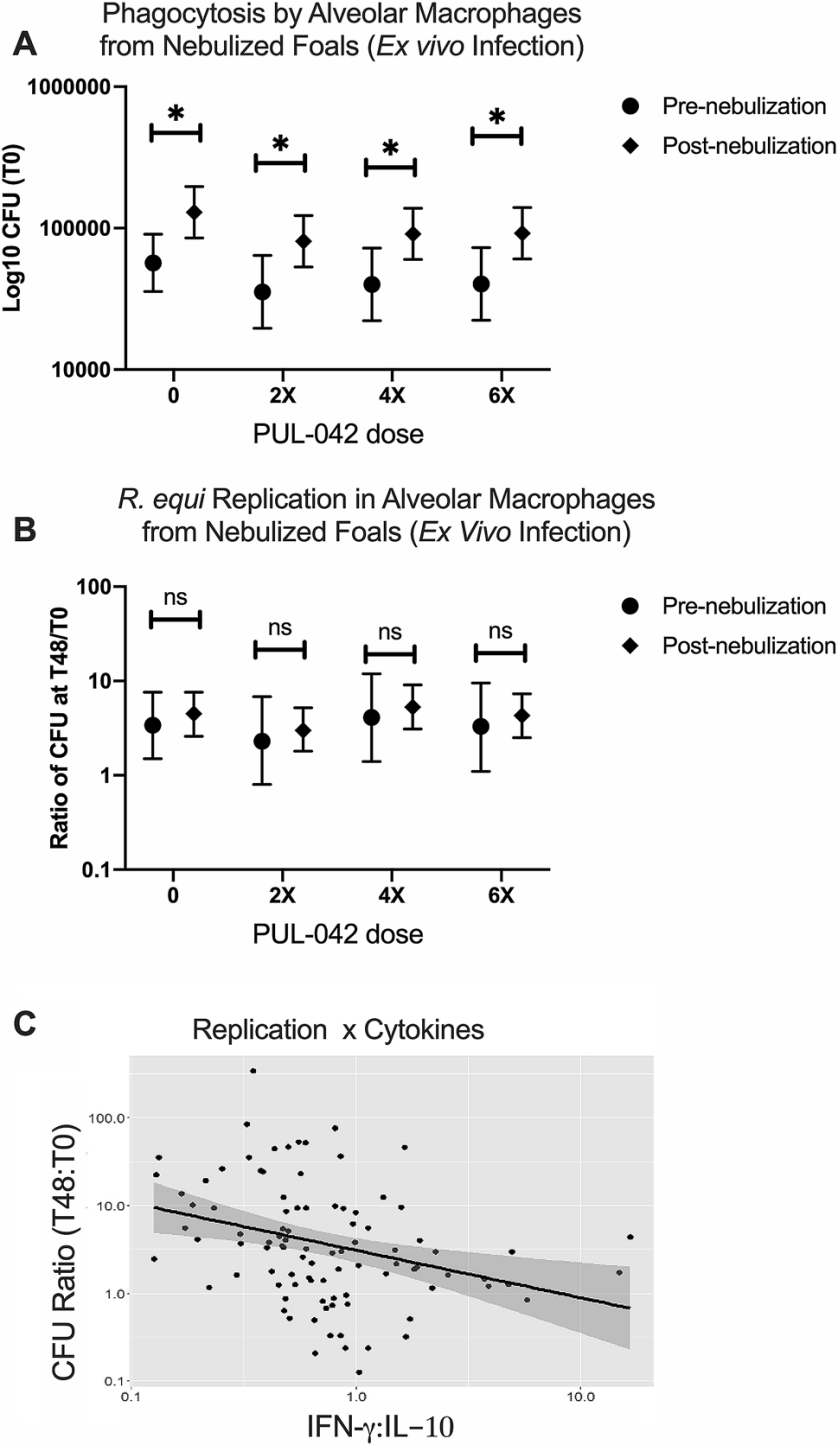
Internalization and intracellular killing of *Rhodococcus equi* by alveolar macrophages (AMs) collected via bronchoalveolar (BAL) lavage pre (day 2 after birth) and post (day 22, 1 day after the last nebulization). All 48 foals were nebulized with either glycerol 2.8% (PUL-042 0; n = 12), or varying doses of PUL-042 (2×, n = 12; 4×, n = 12; or 6×, n = 12). No foals were infected in vivo with *R. equi*. Alveolar macrophages were infected with 10^6^ virulent *R. equi* using a multiplicity of infection of 10 bacteria per macrophage (infected) or kept with media only (uninfected control). Cells were then washed and either lysed and diluted immediately (T0) for bacterial determination, or cultured for 48 h on 5% CO2 and then lysed and diluted for CFU counts (T48). There were not significant effects of treatment or age in either phagocytosis ((**A**); T0) or replication ((**B**); ratio of T48:T0). (**C**) CFU ratio (T48:T0) vs IFNg:IL-10. The CFU ratio (lower ratio = greater intracellular killing) was significantly (P = 0.0075) and negatively associated with the ratio of IFN-g:IL-10 expression ratios (infected/uninfected). The log10 CFU ratio decreased by 10^(−0.47397)^ (95% CI, 10^(−0.8149639)^ to 10^(−0.132983)^). R-value − 0.4740.

Phagocytosis of virulent *R. equi* by AMs from nebulized foals (Fig. 1A) significantly increased with age in animals of all groups at age 22 days compared with age 2 days (T0; Fig. 5A). Nebulization with PUL-042 did not increase phagocytosis in AMs (Fig. 5A), nor did it improve killing of *R. equi* intracellularly (ratio of T48/T0; Fig. 5B). Because the balance of pro- and anti-inflammatory cytokines is more important than their individual concentrations, we examined the ratio of stimulated IFN-γ expression (ratio of infected to uninfected cells) and IL-10 expression (ratio of infected to uninfected cells), and found that it was associated with killing of *R. equi* (Fig. 5C; R value − 0.4740).

### Nebulization with PUL-042 reduces severity of pneumonia

**Figure 6:**
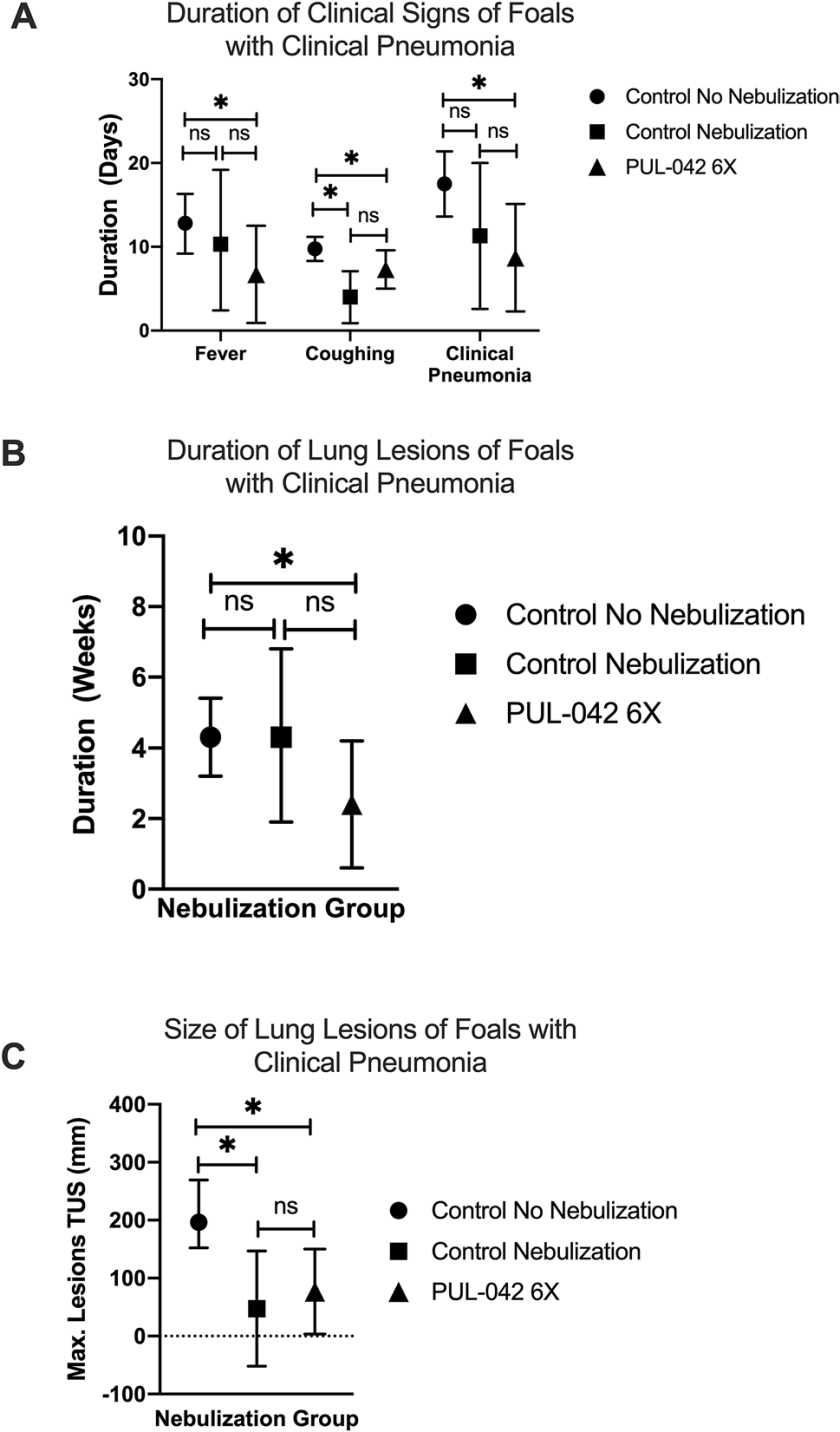
Clinical parameters of foals not nebulized (group control no nebulization; n = 12), nebulized with either PUL-042 6× (139.8 µg Pam2:204 µg ODN diluted in 2.8% glycerol; group PUL-042 6×; n = 7) or glycerol 2.8% (group control nebulization; n = 3) before and after infection with 10^6^ virulent *R. equi* intrabronchially at 28 days of age. (**A**) Non-nebulized foals showed longer duration of clinical signs of pneumonia, coughing, and fever when compared to foals receiving PUL-042 6×. (**B**) Nebulization with PUL-042 6× had shorter duration of presence of abscesses in their lungs upon thoracic ultrasound examination when compared to both control groups, although only group control no nebulization was significantly longer. (**C**) Foals that did not receive any nebulization showed larger sum of ultrasonographic lesions than foals that were nebulized with either PUL-042 6× or glycerol 2.8%. Mean is represented by the symbol (circle for control no-nebulization; square for control nebulization; and triangle for PUL-042 6×) and 95% confidence interval (whiskers).

After we ensured the safety of nebulization of PUL-042 in 48 foals, and its ability to modulate the immune responses of AMs following ex vivo infection with *R. equi* through both pro- and anti-inflammatory cytokine production, we examined whether nebulization with PUL-042 before and after intrabronchial infection with live, virulent *R. equi* decreased the severity of the resultant pneumonia in foals (Fig. 1B). All foals in all groups experimentally infected were confirmed having *R. equi* clinical pneumonia based on our case-definition: ≥ 2 physical signs, evidence of lung abscessation, and both presence of *R. equi* and cytologic evidence of suppurative pneumonia with presence of degenerate neutrophils in T-TBA samples. Twenty-two additional foals were randomly assigned to 3 groups: control not nebulized, nebulized before and after infection with 2.8% glycerol (PUL-042 diluent), and nebulized before and after infection with PUL-042. Foals that were nebulized with PUL-042 following the infection had a significantly (P < 0.05) shorter duration of clinical signs of pneumonia, coughing, and fever (Fig. 6A), shorter duration of detection (Fig. 6B), and smaller total sum (Fig. 6C) of TUS lesions when compared to foals that did not receive nebulization, but not when compared to foals nebulized with glycerol only.

## Discussion

In this study we found that nebulization of foals with PUL-042 after intrabronchial challenge with *R. equi* reduced the severity of pneumonia in foals. This may be an important finding, because it could be a potential ancillary treatment to antimicrobials for foals with natural *R. equi* pneumonia. It has been hypothesized that HDTs that modulate TNF-α as an adjunct to canonical chemotherapy shorten therapy duration, reduce pathology, and limit tuberculosis relapse in experimental models by decreasing exacerbated inflammation^43^. Additionally, as seen in mice with influenza pneumonia and antiviral therapy^37^, PUL-042 could have a synergistic effect when used in combination with antimicrobials, and it could potentially decrease either the dose of antibiotic or duration of treatment, aiding in the reduction of pressure for development of antimicrobial resistance. In our study, foals were nebulized before clinical pneumonia developed, thus, antimicrobial treatment and nebulization treatments were not contemporaneous. The synergy between concurrent administration of macrolides and PUL-042 in foals with pneumonia merits further evaluation. Surprisingly, we also observed that nebulization procedure with glycerol only (our nebulized control group) was beneficial to reduce severity of disease. Although duration of fever, duration of clinical signs, and duration of lung lesions tended to be higher in the glycerol only group, that difference was not significant. We are not able to explain the reason for this finding. Glycerin compounds are solvents for chemicals in e-cigarettes, and glycerin-based vapors have been shown to increase inflammatory responses in the lungs of mice^44–46^ and to increase cytokine secretion by human AMs and bronchial epithelium^47–50^; however, electronic cigarettes contain glycerol in much higher doses (15–100%) than used in our study (2.8%). Glycerol can stimulate pro-inflammatory cytokine secretion by human monocyte-derived macrophages and cultured airway epithelial cells^51,52^. To the authors’ knowledge, the effects of nebulized glycerol on innate immune responses of equine pulmonary cells have not been investigated, but it is plausible (as suggested by our findings) that nebulization of glycerol solution alone activates innate immune responses, albeit to a lesser extent than the PUL-042. It is possible that glycerol, at low concentrations such as used in this study (2.8%), induces a beneficial inflammatory response. We speculate, however, that the small sample size of the nebulized glycerol vehicle control group precluded us from detecting a significant difference between the PUL-042 and glycerol vehicle. Nevertheless, our results indicate that nebulization with PUL-042 significantly reduced the severity of disease in treated foals.

We measured different cytokines in the supernatant of AMs collected from nebulized foals and infected ex vivo with *R. equi*. Two important pro-inflammatory cytokines, IL-6 and IFN-γ, were only significantly increased in foals treated with PUL-042. There is evidence that young foals are deficient in IFN-γ, and this deficiency has been theorized to explain why foals are more susceptible to *R. equi* pneumonia^7,8^. We have shown previously that a B class CpG-ODN can induce INF-γ in foals, but whether this would translate to a better clinical outcome upon in vivo intrabronchial infection with *R. equi*, has yet to be determined. Alveolar macrophages are the main cell infected with *R. equi*; there is, however, evidence that pulmonary epithelial cells have an important role interacting and responding to intracellular bacteria such as *R. equi* and Mtb^53–55^. There is also evidence that epithelial cells are responsible for inducible resistance conferred by PUL-042 against pneumonia in mice^40^. Responses to PUL-042 by equine epithelial cells have not been evaluated, but they could be potentially important for lung immunity in vivo. Additionally, the concentration of the pro-inflammatory cytokine TNF-α and anti-inflammatory cytokine IL-10 increased in the fluid supernatant of infected AMs from foals from all groups.

We wanted to determine if nebulization with PUL-042 would increase bactericidal capacity of foal AMs. After collecting BAL cells from foals after six aerosol treatments with PUL-042 and infecting them with live *R. equi*, we did not observe either an increase in either phagocytosis or in killing of *R. equi* when compared with foals that received glycerol 2.8% alone. Despite our hypothesis that AMs from foals treated with PUL-042 would kill *R. equi* better than AMs collected from untreated foals, our negative findings were not unexpected. There is evidence that treating macrophages without the presence of epithelial cells is not sufficient to increase killing of *R. equi*^42^, and these cells were not present in our culture system where AMs were infected. Moreover, clearance of *R. equi* can be mediated by other innate immune cells including neutrophils^11,56,57^. We conclude that while nebulization with PUL-042 did not directly appear to impact killing of *R. equi* by AMs, it modulated immune responses to favor improved functional responses that in the pulmonary environment might enhance the capability of pulmonary cells to kill intracellular pathogens.

Because we observed effects of PUL-042 on cytokine production, and these were directly related to bacterial killing^33–35^, we wanted to determine next if cytokine production and bacterial killing were correlated. The CFU ratio (T48/T0; representing bacterial replication) was indeed associated with the ratio of IFN-γ divided by IL-10 in response to infection. The lower the CFU ratio (meaning greater bacterial killing), the higher was the cytokine ratio, suggesting that a balance between pro- and anti-inflammatory cytokines is necessary for intracellular killing of pathogens.

We also showed in this study that nebulization of PUL-042 in foals resulted in no adverse effects such as lacrimation, increased respiratory effort, or nasal discharge in any of the 43 foals nebulized multiple times with PUL-042. We also did not observe any signs of systemic inflammatory responses induced by PUL-042, because concentrations of pro- or anti-inflammatory cytokines or SAA did not increase in serum of foals after nebulization with any nebulization treatment. We found that foals had significantly higher concentrations of SAA at age 2 days than age 28 days, regardless of the treatment group. Serum amyloid A is an acute-phase protein that rapidly increases with pneumonia or when there is an inflammatory stimulus^58^. To our knowledge, there is no report evaluating in foals either the age-related change in SAA during the first month after birth, nor following nebulization. Possible explanations for our findings are that 2-day-old foals have higher SAA after ingesting colostrum, or that nebulization procedure per se induced inflammation. There is no evidence that nebulization increases SAA, and because we do not have age-matched control foals that were not nebulized in the ex vivo study, we cannot ascertain what caused the age-related reduction in SAA.

We observed a significant decrease in the % of neutrophils and increase in the % of macrophages in BAL fluid from nebulized foals, irrespective of treatment, while the % of lymphocytes remained unchanged. Hostetter et al. (2017) also observed that the % of neutrophils decreased and the % macrophages increased in the first month when compared to 1-week-old foals, but the differences were not significant^59^. In our experience, in the first 3 days after birth the % of neutrophils are higher than in 1 week-old-foals, which would explain the discrepancy between the two studies.

An increase in the % of neutrophils in BAL fluid would have indicated a transient local inflammation following nebulization, because neutrophil infiltration in the lungs is considered a marker for inflammation. In the case of infection with *Streptococcus pneumoniae* (Spn) in mice, however, protection against lethal Spn pneumonia conferred by a nebulized Spn lysate was independent of neutrophil recruitment to the lungs^60^. We do not have evidence in our study that lung inflammation occurred following nebulization, other than an increased in TNF-α in BAL fluid of foals receiving PUL-042 2× and 4× but not 6×. We consider this to be a positive feature of the treatment in newborn foals because exacerbated inflammation can worsen the severity of pneumonia. All other cytokines remained unchanged in the BAL fluid of nebulized foals.

We recognize that additional studies are necessary to optimize the timing and dose for potentially stronger effects of PUL-042 as well as to overcome anatomical challenges to nebulization of horses, such as their longer tracheas, obligate nasal breathing, etc. Nevertheless, our findings that PUL-042 stimulates innate immunity of foals are promising considering the naivety/immaturity of their immune system in the first month after birth^4,5,7,8,31^. It is important to note that *R. equi* experimental infections (including the challenge model used in our study) usually induce a more severe pneumonia than observed in natural infection, likely due to the higher number of infecting organisms used in experimental challenges. Thus, it is possible that nebulization with PUL-042 might be more effective against natural infection with lower doses. Nevertheless, our results show potential for improving neonatal immune responses against bacterial respiratory pathogens, and reducing severity of clinical pneumonia following intrabronchial challenge with *R. equi*.

## Material and methods

### Ethics statement

All methods were performed in accordance with the relevant guidelines and regulations. The study was carried out in compliance with the ARRIVE guidelines. All procedures for this study were reviewed and approved by the Texas A&M University Institutional Animal Care and Use Committee (IACUC), Animal Use Protocol (AUP) # 2014-0358 CA, 2018-0010, and 2018-0354, and the UGA IACUC AUP# A2019 02-008-YI-AO. The foals used in this study were university-owned, and permission for their use was provided in compliance with the procedures of the referent institution’s IACUC. None of the 70 foals died or were euthanized as a result of this study.

### Experimental design

The design of our project is summarized in a schematic (Fig. 1A,B). We first evaluated the safety and compared the ex vivo effects of three nebulization doses in 48 foals (Fig. 1A). We then conducted a randomized, controlled experiment of the selected dose of PUL-042 to reduce the severity of pneumonia in 22 foals following experimental challenge (Fig. 1B). Investigators with the responsibility for clinical diagnosis were blinded to the nebulization group of the foals. A thoracic ultrasonographic examination (TUS) was performed prior to both nebulization and intrabronchial challenge to ensure that study foals did not develop naturally-acquired *R. equi* pneumonia.

### Bacteria preparation

*Rhodococcus equi* strain 33701+ (ATCC reference virulent VapA^+^ strain; Rockville, MD) was used for ex vivo infection of AMs^61^, and *R. equi* strain EIDL 5–331 (a virulent isolate from a Texas foal that died of *R. equi* pneumonia) was used for in vivo infection of foals^62^. One CFU of the respective strain was inoculated into 50 ml of brain–heart infusion broth (BHI; BD Biosciences, La Jolla, CA) and shaken for 24 h at 37 °C, and then sub-cultured in 500 ml of BHI broth and shaken for 24 h at 37 °C. The bacterial suspension was centrifuged at 3400×*g* (5810R, Eppendorf AG, Hamburg, Germany) for 20 min at 4 °C. The BIH broth supernatant was discarded, the pellet resuspended in 100 ml of PBS, centrifuged, and the PBS supernatant discarded. This procedure was repeated twice. The pellet was then resuspended in PBS, and the concentration of bacteria was determined spectrophotometrically (Genesys 20, Thermo Scientific, Waltham, MA, USA). For preparation of infectious inocula, bacterial suspensions were diluted to reach the desired bacterial concentration. Strains were confirmed to be virulent (*VapA* positive) by polymerase chain reaction (PCR) in each inoculum before infection^63^.

### Nebulization of foals for ex vivo experiments

Forty-eight Quarter-Horse foals were used to evaluate the safety and the ex vivo effects of nebulization with PUL-042 on AMs (Fig. 1A). All foals were monitored daily by a veterinarian (AIB, NDC, SG), had age-appropriate results of complete blood count (CBC) on day 2 of age, and adequate transfer of passive immunity assessed by a commercially-available semi-quantitative immunoassay for serum concentration of total IgG (SNAP test; IDEXX, Inc., Westbrook, ME, USA). All treatments were administered using a commercially available foal nebulizer (Flexineb Portable Equine Nebulizer System-Foal Size, Nortev LTD, Gallaway Ireland). Each corresponding treatment was diluted with 2.8% glycerol (Sigma-Aldrich, St. Louis, MO) in sterile water and administered to the foals three times per week for two weeks (total of 6 nebulizations), starting on day 9 after birth. This frequency was based on previous studies of administration of PUL-042 in laboratory animals^64^. Previous preclinical studies projected a therapeutic dose of 23.2 µg Pam2 and 34 µg ODN; the doses selected were 2-, 4-, and 6-fold higher (termed PUL-042 2×, PUL-042 4×, and PUL-042 6× dose, respectively)^64,65^. Foals in both participating institutions (Texas A&M University and University of Georgia; 24 foals in each) were randomly assigned in four groups (Fig. 1A):

1. control PUL-042 0 (nebulized with 2.8% glycerol; n = 12);
2. PUL-042 2X (PUL-042 [46 µg Pam2:68 µg ODN], Pulmotect, Inc.; n = 12);
3. PUL-042 4X (PUL-042 [92.8 µg Pam2:136 µg ODN], Pulmotect, Inc.; n = 12); and,
4. PUL-042 6X (PUL-042 [139.8 µg Pam2:204 µg ODN], Pulmotect, Inc.; n = 12).

### Nebulization of foals for in vivo infection

For the experimental infection study (Fig. 1B), a different group of foals (n = 22) was used. Ten foals were nebulized multiple times pre- and post-challenge using the same nebulizer described above: seven foals were nebulized with PUL-042 6× (139.8 µg Pam2:204 µg ODN diluted in 2.8% glycerol) and three foals were nebulized with 2.8% glycerol. Twelve foals were not nebulized and served as our control non-nebulized group. Our study was powered with the following assumptions: that PUL-042 would reduce the duration of treatment by 7 days, with a standard deviation of 4 days, power of 80%, significance level of 0.05. Calculations indicated we would need seven foals, respectively, but we included an extra 5 control foals to add power. Post hoc, we added three foals to ensure that effects of nebulization of the vehicle alone. All 22 foals were born in the same year and were housed, managed, and infected similarly and contemporaneously with the other foals.

### Broncho-alveolar lavage (BAL)

For the ex vivo study (Fig. 1A), a BAL was performed on foals at ages 2 and 22 days, with an interval of 1 week between the first BAL (at age 2 days) and the first nebulization; the second BAL was performed 1 day after the last nebulization. The BAL procedure and processing of BAL fluid was performed as previously described^61,66^. Briefly, a BAL catheter (Jorgensen Labs, Colorado) was passaged down the trachea and lodged into a bronchus and the air cuff was inflated. Two 60-ml syringes containing 120 ml of sterile saline solution (0.9% NaCl) were infused through the catheter and immediately aspirated, then the process was repeated 60-ml at a time until a total of 360 ml of saline was infused. A 3-ml aliquot of the BAL fluid containing cells was separated for differential cytology by the respective diagnostic laboratories at each institution. BAL cells were centrifuged at 400 ×*g* for 10 min, washed once with PBS, and then re-suspended in MEM-α (Lonza, Walkersville, MD) containing 10% heat-inactivated horse serum (Gibco, Gaithersburg, MD), 1% l-glutamine–penicillin–streptomycin solution (PSG; Sigma Aldrich, St. Louis, MO) and amphotericin B (25 μg/ml; United States Pharmacopeia, Rockville, MD). Cells were then counted using a cell counter (CellometerAuto T4, Nexelom Bioscience, Lawrence, MA) using trypan blue (Sigma Aldrich, St. Louis, MO) and diluted to a concentration of 1 × 10^6^ live cells/ml. Serum and BAL fluid were analyzed for pro- and anti-inflammatory cytokine (TNF-α, IL-1 α, IL-1 β, IL-6, and IL-10) concentrations using enzyme-linked immunosorbent assay (ELISA) kits performed according to manufacturer’s instructions (Table 1).

**Table 1:** ELISA commercial kits and manufacturers used in the study.

Sample	Cytokine	Manufacturer	Location	Catalog #
Culture supernatant	IFN-γ	Mabtech, Inc	Cincinnati, OH	3117-1H-20
BAL fluid	IL-1α	Kingfisher Biotech, Inc	St. Paul, MN	DIY0698E-003
BAL fluid	IL-1β	Kingfisher Biotech, Inc	St. Paul, MN	DIY0699E-003
BAL fluid, serum, culture supernatant	IL-6	R&D Systems, Inc	Minneapolis, MN	DY1886
BAL fluid, serum, culture supernatant	IL-10	R&D Systems, Inc	Minneapolis, MN	DY1605
Culture supernatant	IL-17α	Kingfisher Biotech, Inc	St. Paul, MN	VS0364E-002
Serum	SAA	Kamiya	Seattle, WA	KT-660
BAL fluid, serum, culture supernatant	TNF-α	R&D Systems, Inc.	Minneapolis, MN	DY1814

*BAL* broncho-alveolar lavage, *IFN-γ* interferon-gramma, *IL-1α* interleukin-1 alpha, *IL-1β* interleukin-1 beta, *IL-6* interleukin-6, *IL-10* interleukin 10, *IL-71α* interleukin-17 alpha, *SAA* serum amyloid A, *TNF-α* tumor necrosis factor-alpha.

### Intracellular *R. equi* killing assay

Macrophage intracellular killing assays were performed as previously described^61,66^. One ml of cell suspension was placed in triplicates in wells of two 24-well plates: the first plate was used for CFU determination immediately after 40 min of infection to represent phagocytosis (T0) and the second plate was used for CFU determination after 48 h of incubation to represent bacterial survival or replication (T48). Cells were incubated overnight in supplemented MEM-α at 37 °C with 5% CO2. The next morning, cells were washed with warm PBS and 1 × 10^6^ *R. equi* (ATCC 33701+ strain, multiplicity of infection [MOI] of 10) in 1 ml of fresh MEM-α containing 10% non-heat-inactivated horse serum without amphotericin B and PSG were added, and the infected cells were incubated for 30 min at 37 °C with 5% CO2. Assay controls consisted of wells with media only. Following infection, cells from T0 plates were lysed with 1 ml/well of distilled water, and the suspension was serially diluted and plated out onto BHI agar plates to determine the concentration of live bacteria. Cells from T48 plates were washed with warm PBS, received 2 ml MEM-α containing 10% heat-inactivated horse serum, amphotericin B, and amikacin (8 μg/ml, United States Pharmacopeia, Rockville, MD), and were incubated for 48 h at 37 °C with 5% CO2. After 48 h, cells were submitted to the same lysing procedure as T0 plates. The mean of triplicate wells was used for data analysis. Supernatant of cultured BAL cells were analyzed for pro- and anti-inflammatory cytokine (IL-1α, IL-6, IL-17, IL-10, IFN-γ, and TNF-α) concentrations using ELISA kits performed according to manufacturer’s instructions (Table 1).

### In vivo infection of foals

For transendoscopic infection^11,62^, foals were sedated using intravenous (IV) injection of xylazine hydrochloride (0.5 mg/kg; Vedco, St. Joseph, MO) and IV butorphanol tartrate (0.02 mg/kg; Zoetis, Florham Park, New Jersey). An aseptically-prepared video-endoscope with outer diameter of 9 mm was inserted via the nares into the trachea and passed to the bifurcation of the main-stem bronchus. A 40-ml suspension of virulent EIDL 5–331 *R. equi* containing approximately 1 × 10^6^ viable bacteria was administered transendoscopically, with 20 ml infused into the right mainstem bronchus and 20 ml into the left mainstem bronchus via a sterilized silastic tube inserted into the endoscope channel. The silastic tube was flushed twice with 20 ml of air after each 20-ml bacterial infusion. Foals and their mares were housed individually in stalls and separately from other mare and foal pairs for one week following experimental infection. After 1 week, these mare/foal pairs were transferred back to their original pasture.

### Clinical monitoring

Clinical monitoring was performed as previously published^11,62^. Physical examination of foals was performed daily from birth by a veterinarian to observe the presence of any adverse effects of the nebulization and signs of exacerbated inflammation of the lungs. The following parameters were recorded: (1) rectal temperature, heart rate, and respiratory rate; (2) tracheal and thoracic auscultation for the presence of abnormal lung sounds (crackles or wheezes, evaluated for both hemithoraces); (3) presence of coughing or nasal discharge; (4) respiratory effort; and, (5) attitude (active or lethargic, including posture and frequency of suckling). For the ex vivo study using non-infected foals, physical examination was performed once daily and continued until 1 week after the second BAL. For infected foals in the in vivo study, physical examination was performed twice daily following infection to detect clinical signs of pneumonia until 12 weeks of age, or until resolution of pneumonia if the foal remained ill after 12 weeks of age. As previously described^11,62^, animals were also subjected to weekly TUS to identify evidence of peripheral pulmonary consolidation consistent with *R. equi* pneumonia. Foals were considered to have clinical pneumonia if they demonstrated ≥ 2 of the following clinical signs: coughing at rest; dull attitude (reluctance to rise, lethargy, increased recumbency); rectal temperature > 39.4 °C; respiratory rate ≥ 60 breaths/min; or, increased respiratory effort (manifested by abdominal lift and nostril flaring) plus ultrasonographic evidence of pulmonary consolidation with a maximal diameter of ≥ 2.0 cm, positive results of culture of *R. equi* from transendoscopic tracheobronchial aspirate (T-TBA) fluid, and cytologic evidence of septic pneumonia from T-TBA fluid. The T-TBA was performed by washing the tracheobronchial tree with 20 ml of sterile PBS solution delivered through a triple-lumen, double-guarded sterile tubing system (MILA International, Inc. Erlanger, KY, USA), and then aspirating fluid from the tracheobronchial tree through the same catheter system. A T-TBA was performed at the time of onset of clinical signs of pneumonia, and in all foals at the termination of the study at age 12 weeks for all foals (end of study).

The outcomes of the in vivo study were the duration of days meeting the case definition of *R. equi* pneumonia, the number of days with lesions detected on TUS, and sum of the total maximum diameter (TMD) of ultrasonography lesions over the study period^11,62^. The TMD was determined by summing the maximum diameters of each lesion recorded in the 4th to the 17th intercostal spaces from each foal at every examination; the sum of the TMDs is an index that incorporated both the duration and severity of lesions. Foals diagnosed with *R. equi* pneumonia were treated with a combination of clarithromycin (7.5 mg/kg; PO; q 12 h) and rifampin (7.5 mg/kg; PO; q 12 h) until both clinical signs and thoracic ultrasonography lesions had resolved^11,62^.

### Data analysis

ELISA and cytology data were analyzed using mixed-effects modeling with the outcome variable being immune/inflammatory parameters of interest (e.g., the proportion of neutrophils or concentration of cytokines in BAL fluid or serum); treatment groups, times (2 days or 22 days), and their interaction terms were modeled as fixed independent variables; individual foal was included as a random-effect term. The proportional killing capacity of *R. equi* by AMs also were included as an outcome variable. Model fit was assessed by inspection of diagnostic residual plots. All analyses were performed using R software (version 3.5.1, R Foundation for Statistical Computing, Vienna, Austria), and using a significance level of P < 0.05.

For the clinical data, continuous variables were compared among the 3 groups with regard to post-challenge treatment: (1) no nebulization after; (2) nebulization after with glycerol; and, (3) nebulization after with PUL using a generalized linear model with a Gaussian link. Categorical data were summarized as tables and analyzed using Fisher’s exact tests. Analysis was performed using R software with a significance level of P < 0.05.
